# The relationship between drive to thinness, conscientiousness and bulimic traits during adolescence: a comparison between younger and older cases in 608 healthy volunteers

**DOI:** 10.1186/1744-859X-12-34

**Published:** 2013-10-31

**Authors:** Concetta De Pasquale, Maria Luisa Pistorio, Eleonora Tornatore, Domenico De Berardis, Michele Fornaro

**Affiliations:** 1 Department of Education Science, University of Catania, Catania 95125, Italy; 2 National Health Service, Teramo 64100, Italy

**Keywords:** Adolescence, Personality traits, Bigfive, Conscientiousness, Thinness, Bulimia nervosa

## Abstract

**Background:**

Adolescence represents one of the critical transitions in the life span and is characterized by a tremendous pace in growth and change that is second only to that of infancy. Both biological and psychological changes occurring during early adolescence may also influence the definition of subsequent late adolescence or early adulthood physiological or (psycho)-pathological features, including bulimia nervosa (BN) whenever occurring. Therefore, a pre-emptive assessment of suggestive psychological traits, including bulimic ones, during early and late years of adolescence, is recommended and represents the goal of the present study.

**Methods:**

Six hundred and eight healthy volunteers attending mid- or high school, aged 14–19 years, were consecutively enrolled at multiple sites in Eastern Sicily, Italy. A systematic psychological assessment was performed, including McCrae and Costa' BigFive, the Eating Disorders Inventory (EDI), Bisantis's Assertivity test and the Liebowitz Social Anxiety Scale for Children and Adolescents. Demographic and general characteristics, including the body mass index, were also recorded. Based on hierarchical considerations, cases were then divided into ‘younger’ (‘early’ years, 14–16) and ‘older’ (‘late’ years, 17–19) adolescents.

**Results:**

Upon descriptive and Pearson's correlation analyses, the following EDI constructs ‘drive to thinness’ and ‘bulimia’ scored significantly higher (both *p* = <.001) in ‘early’ vs. ‘late’ cases. Conversely, BigFive ‘conscientiousness’ was higher in older subjects vs. early cases (*p* = <.003). As expected, ‘drive to thinness’ positively correlated with BN both in early (*r* = .31) and late (*r* = .50) cases. In the ‘late’ group, age correlated with conscientiousness (*r* = .206) while BN correlated with drive to thinness (*r* = .505); finally, a negative correlation was observed with regard to consciousness and BN (*r* = −.19).

**Conclusions:**

Despite intrinsic methodological limits, our preliminary findings confirm that the transition between early and late years of adolescence is a critical phase of life span, with the consolidation of ‘conscientiousness’ eventually playing a protective role towards the onset of bulimic traits. If confirmed by replication studies, ideally providing long-term follow-ups too, an early acknowledgement of bulimic traits may play a major predictive role for subsequent BN, ultimately contributing to more effective pre-emptive interventions as well.

## Background

The World Health Organization identifies adolescence as the period in human growth and development that occurs after childhood and before adulthood, from ages 10 to 19. It represents one of the critical transitions in the life span and is characterized by a tremendous pace in growth and change that is second only to that of infancy. Biological processes drive many aspects of this growth and development, of both somatic and psychological nature, with the onset of puberty marking the passage from childhood to adolescence. The biological determinants of adolescence are fairly universal; however, the duration and defining characteristics of this period may vary across time, cultures and socioeconomic situations [1].

On the contrary, what is broadly accepted, is the clinical wisdom supported by literature evidence indicating many psychiatric disorders including disruptive disorders, anxiety disorders and depressive disorders, having a common age of onset at childhood or adolescence [2,3]. For some of these disorders, including bulimia nervosa (BN), the typical age of onset may fall within the early years of adolescence (≤15 years) and late adolescence (17–21 years) [4,5]. Yet, ‘clear cut’ onsets of BN, especially during early adolescence, are of difficult definition, therefore shifting the interest of clinicians also towards clinically suggestive psychological traits, which may direct or anticipate the onset of clinically meaningful BN [6]. Specific bulimic traits may influence the dietary pattern and/or familial, social (scholastic) habits of ‘healthy’ subjects, sometimes since the early years of adolescence, often going greatly under-recognized even though they could find expression as full-threshold, clinically meaningful, psychopathological personality, or axis-I psychiatric disorders (to onset at late adolescence or early adulthood), including BN [7-10]. Bulimic traits are also difficult to detect due to the secrecy of the phenomena and social stigma still associated to BN and its related conducts, leading most of the ‘affected healthy’ adolescents to neglect the burden of their own bulimic conducts [10-12].

As consequence, an early acknowledgment of such bulimic traits is of indisputable relevance, with a special reference towards younger adolescents who may still be in a very sensitive, transitional phase of their own life span and who may therefore preferentially respond to pre-emptive therapeutic interventions, whenever necessary.

Therefore, the aim of this study was to provide a systematic, multi-purpose, psychological assessment of a large sample of healthy adolescents including subjects of ages ranging from 14–19 years in order to explore those personality traits differentially associated to bulimic traits, ideally contributing to a pre-emptive acknowledgment of BN in those vulnerable subjects.

## Methods

### Participants

The subject pool consisted of 608 patients, including both genders, who were enrolled across multiple collaborating mid- and high public schools in Eastern Sicily (Catania, Enna, Ragusa) during May 2008–January 2013. The Kiddie Schedule for Affective Disorders and Schizophrenia, School-Age Children-Present and Lifetime version (K-SADS-PL) [13] was submitted to subjects younger than 18. Subjects aged 18 or 19 were screened by the means of the Structured and Clinical Interview for Axis I Disorder patient module (SCID-I/P) [14]. Both instruments were submitted in order to exclude any psychiatric disorder.

Specifically, the K-SADS is a tool designed for school-aged children of 6–18 years, administered by interviewing the parent(s) and the child/adolescent, estimating ratings, which include parent, child, school and chart. The SCID-I/P is a semi-structured interview conducted by a trained psychiatrist in order to assess the presence of any Diagnostic and Statistical Manual-Fourth Edition (DSM-IV) Axis I Disorder (including sub-threshold ones) in adults samples [15]; personality disorders were assessed (and ruled out) using the Structured and Clinical Interview for Axis II Disorder (SCID-II) scale, which is also a semi-structured tool [16].

Seven clinical psychiatrists and two trained psychologists evaluated the sample through the study. Before subject evaluations, the Cohen'k level of interrater variability across raters was ascertained in order to ensure reliable comparisons. All subjects (or their parents in case of minors) provided a written informed consent after procedures had fully explained by a trained MD or PhD in the presence of both parents and/or teachers, after procedures had approved by respective local Ethical Committees. The demographic data and non-psychiatric medical history records, including the body mass index (BMI), relied on self-report interviews vouched by the parents. The psychological assessment included the following instruments: the McCrae and Costa' BigFive [17], a 44-item self-report inventory designed to measure the Big Five dimensions, consisting of short phrases with relatively accessible vocabulary; the Eating Disorders Inventory (EDI), a self-report questionnaire used to assess the presence of eating disorders (EDs) (submitted alongside with the SCID-I module for EDs), (a) Anorexia nervosa both restricting and binge-eating/purging type; (b) Bulimia nervosa; and (c) eating disorder not otherwise specified including binge eating disorder (BED), including 64 questions, divided into eight subscales [18,19], Bisantis's Assertivity test, a 20-item, multiple choice questionnaire aimed at measuring one's assertivity, defined as ‘the ability to recognize and express his/her own emotions, to claim his/her own rights, and to manifest his/her own needs, preferences, desires and critics’ [20] and the Liebowitz Social Anxiety Scale for Children and Adolescents (LSAS-CA-SR), a 24-item scale descripting 12 social interactions and 12 performance situations [21].

### Statistical analysis

Descriptive and inferential statistics were performed using the IBM SPSS v.20 software for Windows. Based on methodological considerations derived by previous literature evidences [8,10], the sample was divided into ‘early’ (age 14–16 years) vs. ‘late’ (17–19 years) groups in order to compare both demographic and psychological characteristics. Since data followed a normal distribution, as assessed by the Kolmogorov-Smirnov test, parametric procedures were adopted across the analyses, including chi-square (for categorical variables) and independent-sample *t* tests (for continuous variables), as well as Pearson's correlation. Data were reported using ± SD means. *P* values ≤ 0.05 were considered significant.

## Results

### Interrater variability

The preliminary interrater variability for the raters for the diagnosis made with the EDI and the BigFive tools was Kappa .639 (*p* = .001), 95% CI (−.0710, .1038).

Additional measures of interrater variability for other scales alternatively ranged from .72 (‘substantial agreement’) to.83 (‘almost perfect agreement’) among raters.

### Major findings

Basic demographic and psychological features of the sample have been reported in Table 1.

**Table 1:** Demographic and clinical features of the subjects included in the study

**Study subjects (*n*** **= 608)**	**Early adolescence (age 14–16)** ***N*** **= 341 (56.08%)**	**Late adolescence/early adulthood (age 17–19)** ***N*** **= 267(43.91%)**	***T *** **or *χ*** **2(df = 2)**	***p***
Demographics				
Age in years, mean ± sd	15.03 ± .81	17.74 ± .76	42.100(606)	<0.001
Sex F/M, *n*(%)	258(75.70 %)/83(24.3%)	178(66.70 %)/89(33.3%)	5.970(1)	ns
BMI, mean ± sd	21.13 ± 3.26	21.89 ± 3.34	2.580(503)	ns
Bisantis' assertiveness test, mean ± sd				
Passive (range)	41.76 ± 8.24(20–62)	42.24 ± 9.26(15–62)	.674(606)	ns
Aggressive (range)	21.92 ± 5.32(6–39)	22.19 ± 5.09(1–32)	.631(606)	ns
Assertive (range)	63.63 ± 9.12(36–85)	64.29 ± 10.40(18–84)	.831(606)	ns
Eating Disorder Inventory (EDI), mean ± sd				
Drive to thinness (range)	5.64 ± 5.62(0–21)	4.21 ± 4.25(0–19)	−3.465(606)	.001
Bulimia (range)	3.46 ± 3.72(0–16)	2.54 ± 3.00(0–14)	−3.308(606)	.001
Body dissatisfaction (range)	7.99 ± 6.49(0–31)	7.43 ± 6.08(0–26)	−1.092(606)	ns
Ineffectiveness (range)	4.67 ± 4.64(0–34)	4.42 ± 4.56(0–26)	–.654(606)	ns
Perfectionism (range)	5.21 ± 3.59(0–16)	5.31 ± 3.22(0–15)	.353(606)	ns
Interpersonal distrust (range)	5.74 ± 3.49(0–17)	5.23 ± 3.48(0–18)	−1.801(606)	ns
Interocept awareness (range)	6.93 ± 5.78(0–28)	5.75 ± 5.04(0–26)	−2.634(606)	ns
Maturity fears (range)	7.96 ± 3.90(0–19)	8.12 ± 4.39(0–22)	.478(606)	ns
EDI (total score) (range)	47.53 ± 23.85(1–142)	42.66 ± 20.85(8–147)	−2.637(606)	ns
Liebowitz' Social Phobia Scale (LSPS), mean ± sd				
Fear of relationship sub-scale (range)	14.87 ± 7.32(0–49)	13.91 ± 6.35(0–34)	−1.708(606)	ns
Avoidance of relationship sub-scale (range)	13.87 ± 6.54(0–36)	13.02 ± 6.37(0–30)	−1.595(606)	ns
Fear of performance sub-scale (range)	28.74 ± 12.93(1–80)	26.93 ± 11.44(0–63)	−1.801(606)	ns
Avoidance of performance sub-scale (range)	12.47 ± 7.01(0–33)	11.82 ± 6.21(0–30)	−1.202(606)	ns
Total fear sub-scale (range)	12.25 ± 6.34(0–33)	11.54 ± 6.05(0–31)	−1.405(606)	ns
Total avoidance sub-scale (range)	24.68 ± 12.50(0–66)	23.36 ± 11.17(0–61)	−1.357(606)	ns
LPSP total score (range)	53.18 ± 24.01(2–139)	50.15 ± 21.36(0–121)	−1.620(606)	ns
BigFive, mean ± sd				
Extraversion (range)	81.88 ± 10.41(58–113)	78.25 ± 46(43–106)	−2.628(226)	ns
Agreeableness (range)	77.69 ± 6.99(57–59)	75.39 ± 9.33(45–95)	−2.095(226)	ns
Conscientiousness (range)	77.86 ± 9.90(53–101)	81.61 ± 8.92(57–101)	3.005(226)	.003
Neuroticism (range)	61.37 ± 12.50(39–93)	64.36 ± 11.27(33–93)	1.900(226)	ns
Openness to experience (range)	76.76 ± 8.14(61–101)	77.74 ± 9.68(55–101)	.819(226)	ns

The psychological features showing a significant difference across early vs. late years adolescents were then further analysed using Pearson's correlation test. As reported in Table 2, ‘drive to thinness’ and ‘bulimia’ scores were significantly higher (both *p* = <.001) in early vs. late cases. Conversely, ‘conscientiousness’ was higher in older subjects vs. early cases (*p* = <.003). ‘Drive to thinness’ was positively correlated with bulimia both in early (*r* = .31) and late (*r* = .50) cases. In the ‘late’ group, age correlated with conscientiousness (*r* = .206), while bulimia positively correlated with drive to thinness (*r* = .505), showing an opposite correlation trend with consciousness (*r* = −.19).

**Table 2:** Pearson's correlation between significant variables in ‘early’ vs. ‘late’ adolescents

		**Age**	**Eating disorders inventory - drive to thinness**	**Eating disorders Inventory - bulimia**	**BigFive conscientiousness**
Early adolescence	Age	1	−.02	−.10	.13
	Eating disorders inventory drive to thinness	−.02	1	.31**	−.04
	Eating disorders inventory bulimia	−.10		1	−.12
	BigFive conscientiousness	.13			1
Late adolescence	Age	1		−.10	.206*
	Eating disorders inventory drive to thinness	−.042	1	.51**	−.085

Not flagged = not significant; *p = <.05 or significant; **p = <.001 or highly significant; correlation measures refer to those variables already showing a statistically significant difference between early vs. late adolescent groups (as reported in Table 1); additional correlation measures failed to show significant values.

## Discussion

### Limits

There are a number of limits, which should be accounted in the interpretation of the findings of this study and its implications.

While the psychological assessment relied on ‘state-independent’, enduring trait assessed using self-reports instruments, a recall bias may have occurred for other demographic and medical records (including lifetime medications), although the parents and teachers should have verified them. Similarly, the BMI record also relied on self-reported height and weight indicators, which validity tends to be limited nonetheless [22]. Also, some instruments as the EDI would be more suitable for clinical rather than healthy populations, and this may be accounted in the interpretation of results as well. Specifically, while we adopted the adulthood version of the EDI due to the inclusion of some late adolescent/early adult cases, we acknowledge that a children (EDI-C) version of the scale (validated also for use in adolescent healthy subjects) [23] should have been preferred for younger cases. Moreover, the study did not include a medical and/or psychiatric comparison group matched for age range and due to the cross-sectional design of the study; no follow-up information is given about the actual predictive value of bulimic traits on the subsequent onset of BN, which hypothesis largely bases on clinical assumptions. Additionally, the relatively narrow age range (14–19) probably precluded the opportunity to enlighten further potentially statistically significant differences among subgroups. Due to multiple comparisons, data are prone both to type I and type II errors. Finally, no stepwise logistic regression analysis was done at this time stating the preliminary nature of results.

### Clinical implications and future perspectives

In our sample, ‘drive to thinness’, as defined by the EDI, correlated with bulimia both in early and late adolescent cases. The correlation coefficient of drive to thinness with bulimia was weak, and much prudence is needed in interpreting this finding as the expression of a progressive reinforcement of specific pathological behaviors occurring during such sensitive period of life. Nonetheless, if this should be case, this could ideally preclude a further development of the psychiatric and medical consequences that the ‘healthy’ subject should already exhibit during adulthood, although not necessarily (follow-up studies needed) at a full-threshold, clinically meaningful level [10]. Also, a ‘third variable’ bias may limit the legitimacy of the hypothesis made by the present results, urging prudence and, once more, well-designed replication studies.

Concerning ‘conscientiousness’, it showed an inverse correlation trend with bulimic traits only after few years of the adolescence, being therefore more difficult to anticipate in younger adolescents. Nonetheless, it must be remarked that this last correlation seems to be discordant with most of previous literature evidences [24,25] and that correlation coefficients are very low (.19 and .206, respectively), so that a firm conclusion on the matter is discouraged at this time.

## Conclusion

While intrinsic limits related to the design of this study prevent a firm conclusion about the relationship between bulimic traits, conscientiousness and assertivity during early vs. late years of adolescence in healthy subjects, further studies are aimed to shed light on such a sensitive issue, eventually providing long-term follow-ups too.

## Abbreviations

BigFive: McCrae and costa’ BigFive; BMI: Body mass index; BN: Bulimia nervosa; DSM-IV: Diagnostic and statistical manual for mental disorders - fourth edition; EDI: Eating disorders inventory; K-SADS-PL: Kiddie schedule for affective disorders and schizophrenia for school-age children-present and lifetime version; LSAS-CA-SR: Liebowitz social anxiety scale for children and adolescents; SCID-I/P: Structured clinical interview for axis I disorders: patients' edition; SCID-II/P: Structured clinical interview for axis II disorders.

## Competing interests

The authors declare that they have no competing interests.

## Authors’ contributions

CDP conceived the study and enrolled cases. MLP and ET enrolled cases and delivered the psychometric testing. DDB helped in the revision of final version of the text. MF drafted the main text and its subsequent revisions, performing the statistical analysis. All authors read and approved the final manuscript.
